# Comparison of implant primary stability between maxillary edentulous ridges receiving intramembranous origin block grafts

**DOI:** 10.4317/medoral.18732

**Published:** 2013-02-05

**Authors:** Alberto Monje, Florencio Monje, Fernando Suárez, Raúl González-García, Laura Villanueva-Alcojol, Agustín García-Nogales, Pablo Galindo-Moreno, Hom L. Wang

**Affiliations:** 1 D.D.S. D.D.S. D.D.S., M.S., PhD. Graduate Periodontics, Department of Periodontics and Oral Medicine, University of Michigan School of Dentistry, Ann Arbor, MI, USA; 2 MD, PhD. MD, PhD. MD. Oral and Maxillofacial Surgeon, CICOM, Center of Implantology, Oral and Maxillofacial Surgery, Badajoz, Spain; 3 PhD. Department of Mathematics, University of Extremadura, Badajoz, Spain; 4 D.D.S., PhD Professor of Oral Surgery, Department of Oral Surgery, University of Granada, Granada, Spain

## Abstract

Purpose: The purposes of the present study were: to compare the resonance frequency analysis (RFA) values of implant placed in either ramus or calvaria block grafts; and to determine if implant diameter influences RFA implant stability quotient (ISQ) value. Material and Methods: This was a retrospective study that included 16 consecutives healthy patients treated with autogenous onlay block grafts for horizontal bone reconstruction in maxilla. Ten ramus and ten calvaria block graft treated patients were selected and compared. Results: Totally, 59 implants were placed, 35 (59.3%) were placed on the calvaria bone grafts and the remaining 24 (40.7%) were on the ramus bone graft. Of all the implants studied, 13 (22%), 35 (59.3%), and 11 (18.6%) were 10 mm, 11.5 mm and 13 mm in length respectively. Regarding the diameter, 4 (7%) were 3.3 mm, 3 (5%) were 3.5 mm, 20 (34%) were 3.7 mm and 32 (54%) were 4 mm. Mean ISQ value obtained by RFA was 73.06 ± 6.08, being 72.19 ± 6 and 74.47 ± 6.06 for the calvaria and ramus treated group respectively. No significant differences were noted between the two groups (p= 0.154). Implants were pooled and divided by their diameter. Mean ISQ value obtained for 3.3 mm was 80 ± 5.09, while for 4.0 mm was 72.5 ± 7.19. Again, no significant differences were found among the groups (p= 0.138). Conclusion: For RFA ISQ value, the bone graft origins (calvaria or ramus) or implant diameters did not influence the outcome.

** Key words:**Bone augmentation, dental implant, resonance frequency analysis, implant stability.

## Introduction

The term “osseointegration” is defined as “the close contact between bone and implant material in histological observations and, in clinical terms, as the ankylosis of the implant in bone” (1), the absence of mobility represents the primary clinical manifestation of osseointegration. For that, a noninvasive quantitative method for measuring the implant stability was sought out. In 1996, resonance frequency analysis (RFA) was developed and used implant stability quotient (ISQ) as a quantitative unit to assess implant stability (2). It has been reported that RFA ISQ value ranged form 57 to 82 after 1 year of loading (3).

Maxillary bone resorption often results in a ridge that is inadequate for ideal implant placement (4). In order to overcome these challenges, different bone-grafting procedures and materials have been proposed and used in attempts to provide enough height and width for proper implant placement. Many procedures such as sinus lifting (5) or guided bone regeneration (GBR) (6) have been shown to be predictable for bone augmentation. However, autogenous bone block grafting is still considered the “gold standard” in extensive reconstruction of the maxilla (7).

“Creping substitution” is known as the process of bone remodeling, where new bone replaces the necrotic bone, being a longer process in cortical bone (8). Intraoral autogenous grafts have several benefits as well as limitations. It has less amount of bone resorption after healing when compared to endochondral oriented bones (9). In addition, the graft harvesting can be performed in the same surgery and under local anesthesia (10). However, morbidity of their donor site and amount of availability represent the main disadvantages. On the contrary, calvararium is a useful donor site that provides a large amount of intramembranous bone to rebuild the atrophic posterior maxilla. Adding bone substitutes such as particular bone around the autogenous graft to fill the gaps is often performed when doing a big block graft to serve as a scaffold for space maintenance and filler. Generally speaking membrane is not needed when doing the block graft since block can provide the coverage by itself (11,12). However, a collagen absorbable membrane, covering the graft may be suggested, due to its biologic advantages such as the higher stimulation of DNA synthesis over non-resorbables membranes (13).

It has been suggested that the stiffness of the bone might influence RFA (14), hence the purpose of the present study was to compare the ISQ values of intramembranous origin block grafts, either ramus or calvarium, for horizontal bone augmentation in the maxilla. Additionally, the influence of implant diameter upon ISQ value was also evaluated.

## Material and Methods

Sixteen partially edentulous consecutive health patients requiring extensive horizontal bone reconstruction in the maxilla were included in this study. An overall of 20 onlay block grafts were placed. These were harvested either from the ramus (10) or the calvaria (10). Written consent of each subject was signed prior to treatment.

-Surgical protocol

Ramus block graft

Under local anesthesia with intravenous sedation, an incision was performed in the posterior mandible following the external oblique line of the mandible. A full-thickness flap was reflected exposing the lateral aspect of the ramus. Rectangular-shape grafts were harvested. At the recipient site, a mid-crestal incision was performed with intrasulcular and vertical releasing incisions on the adjacent teeth. A full-thickness flap was reflected to expose the recipient area. Ramus block grafts were adapted to the recipient sites and anchored to the alveolar residual bone by two 1.5mm diameter titanium fixation screws (Level One 1.5 Neuro, KLS Martin LP, FL, USA). After achieving stability of the graft, sharp edges of the graft were smoothened using a fissure bur.

- Calvaria block graft

Under general anesthesia with local anesthesia, an incision was performed in the parietal area, parallel to the cranial major axis. Rectangular-shape grafts of the calvaria were marked with a fissure bur and harvested using very gently chisels. A full-thickness flap was reflected to expose the recipient area. Calvaria block grafts were adapted to the recipient sites and anchored to the alveolar residual bone by two 1.5mm diameter titanium fixation screws (Level One 1.5 Neuro, KLS Martin LP, FL, USA). After achieving stability of the graft, sharp edges of the graft were smoothened using a fissure bur.

-Resonance frequency analysis

The technique of RFA measurement followed manufacturer´s recommendations. Basically, a small, precision-crafted metal rod was screwed into the implant side thread. Then, the handheld probe was placed close to the rod first at the midfacial side and the ISQ value, ranging from 1 to 100, was generated and recorded. Immediately after implant placement, implant stability was measured with using Ostell™ Mentor (Integration Diagnostics AB, Göteborg, Sweden). The Smartpeg (Integration Diagnostics AB, Göteborg, Sweden) was attached to the implant with 4-5 Ncm of torque. Two measurements were taken and the averages were calculated to reduce measurements errors.

-Statistics

Statistical package SPSS 13.0 (SPSS Inc., Chicago, IL, USA) and StatSoft, Inc. (2006) STATISTICA (data analysis software system), version 7.1. were used to analyze the data. Descriptive statistical analysis for continuous and categorical variables was performed. Student´s t-test for unpaired samples was used to analyze the influence of implant diameter in primary stability measured by RFA. P value ? 0.05 was considered statistically significant.

## Results

A total of 20 onlay block grafts were placed in the maxilla. These were harvested either from the ramus (10) or the calvarial (10) depending of the amount of graft needed (calvarial > ramus). On them, an overall of 59 implants were placed, being 35 (59.3%) of them placed on calvaria bone grafts ( Table 1) and 24 (40.7%) on ramus bone graft ( Table 2). Mean age of the patients included in the study was 43.8 years old, with a 3:7 male: female distribution. All implants were stable and no mobility was present at the time of prosthesis delivery (4 to 6 months after implant placement).

**Table 1:**
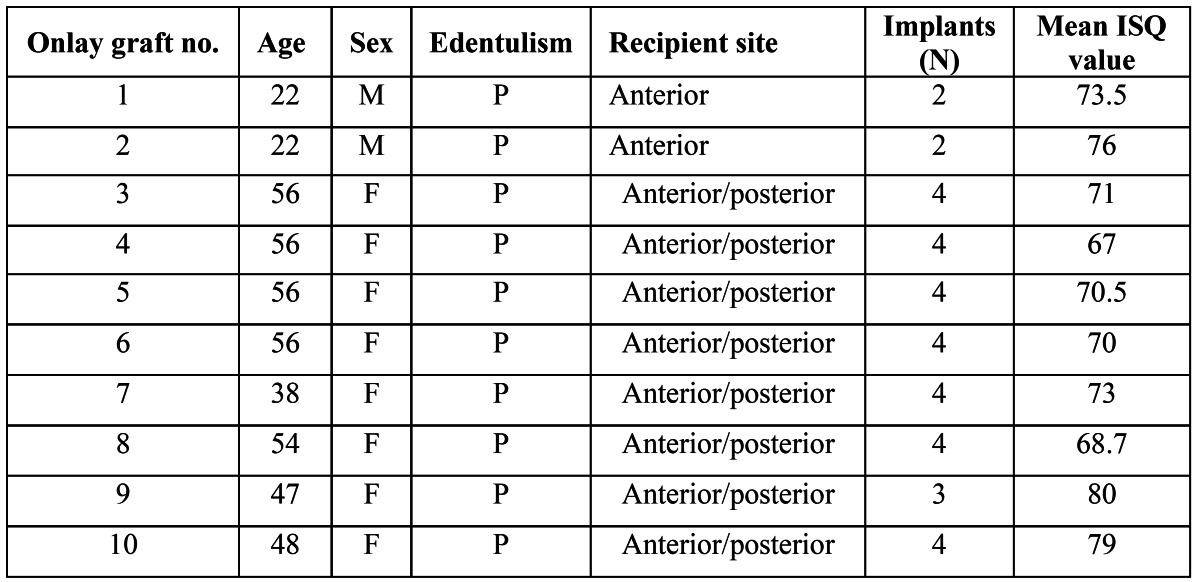
Distribution of the onlay grafts harvested from the calvaria.

**Table 2:**
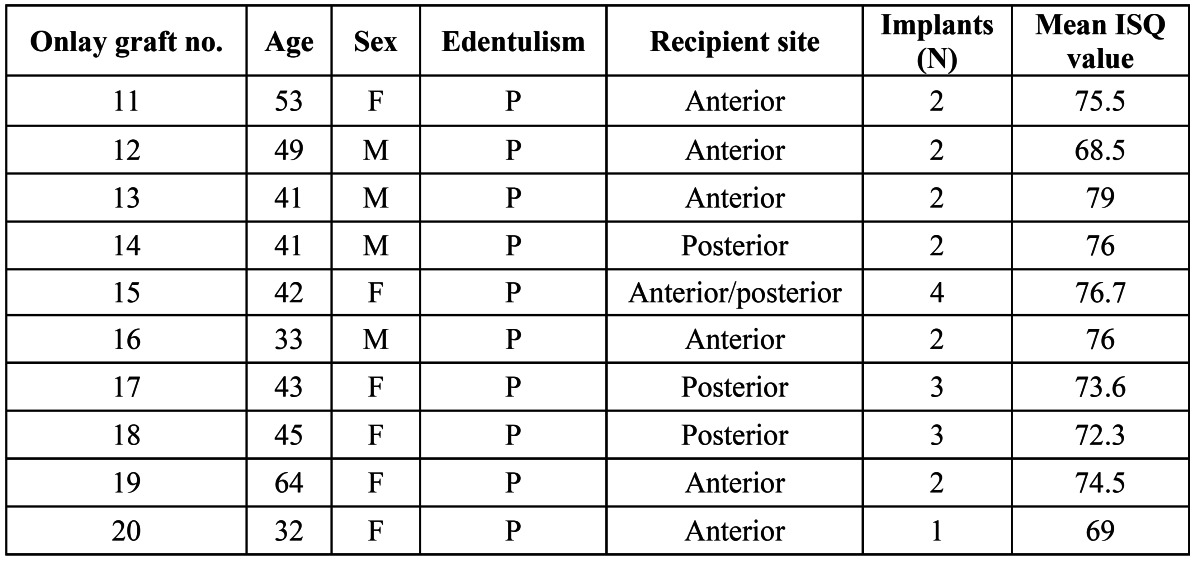
Distribution of the onlay grafts harvested from the ramus.

-Implant length and diameter

**Figure 1:**
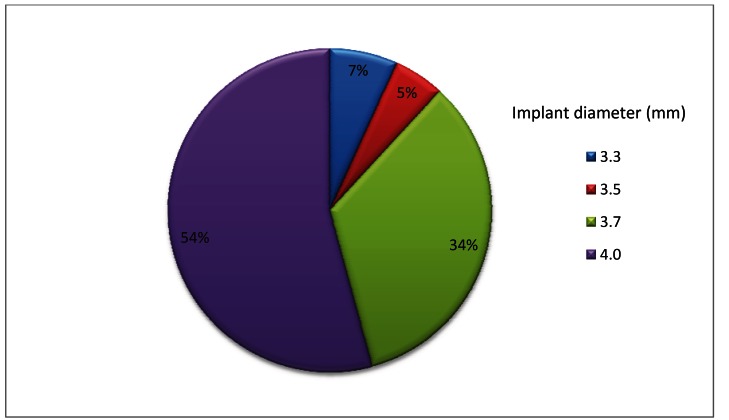
Implant distribution by diameter.

Of all the implants studied, 13 (22%), 35 (59.3%), and 11 (18.6%) were 10 mm, 11.5 mm and 13 mm in length respectively. Regarding the diameter, 4 (7%) were 3.3 mm, 3 (5%), 20 (34%) and 32 (54%) 3.3 mm, 3.5 mm, 3.7 mm and 4 mm, respectively (Fig. 1).

-RFA between both groups

**Figure 2:**
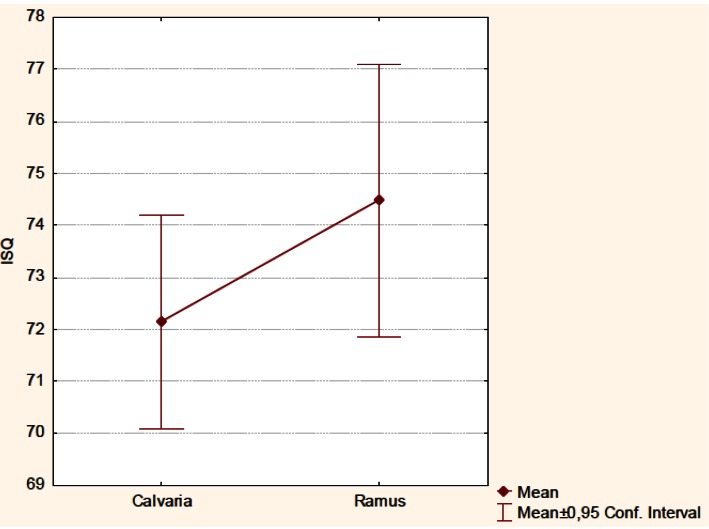
Plot for mean implant stability quotient (ISQ) values from calvaria and ramus groups.

Mean ISQ value was 73.06 ± 6.08, being 72.19 ± 6 for the calvaria group and 74.47 ± 6.06 for the ramus group (Fig. 2). Median ISQ value obtained was 73 for both groups. No significant differences were observed between both groups (p= 0.154) following the application of the Student´s t-test for unpaired data.

RFA between implant diameters

**Figure 3:**
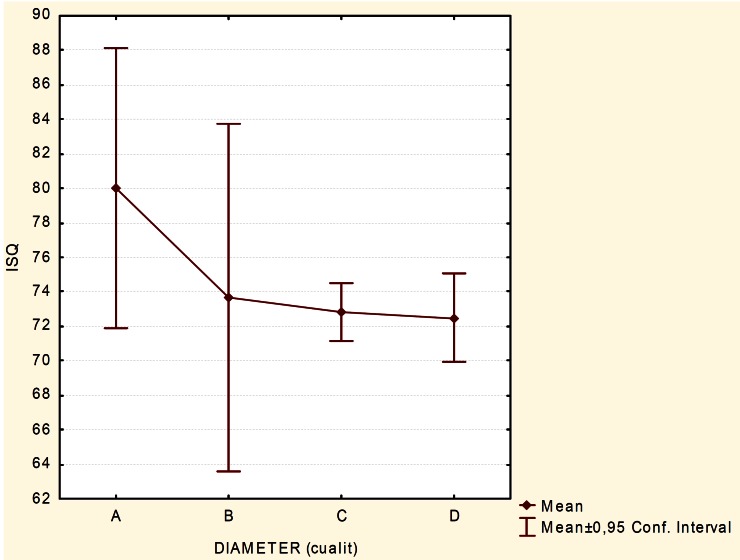
Plot for mean implant stability quotient (ISQ) values for implant diameters analyzed. A. 3.3 mm, B. 3.5 mm, C. 3.7 mm and D. 4.0 mm.

Implants were pooled and divided by their diameter. Mean ISQ value obtained for 3.3 mm (the narrowest implant diameter group) was 80 ± 5.09, while for 4.0 mm (the widest implant diameter group) was 72.5 ± 7.19. Mean ISQ values for 3.5 mm and 3.7 mm implants were 73.66 ± 4.04 and 72.85 ± 7.19, respectively (Fig. 3). Median ISQ values found were 81 for 3.3 mm, 73 for 3.5 mm, 72 for 3.7 mm and 74 for 4 mm implants. Again, no significant differences were found among any groups (p= 0.138). However, there is a trend of higher ISQ value for narrower implants. Nonetheless, it is noteworthy that 3.3 mm and 3.5 mm groups have small sample size (4 and 3 implants, respectively) in comparison with the other two groups analyzed. Hence, precautions should be exercised when interpreting the results of this study.

## Discussion

Primary implant stability plays the most important role in the success of osseointegration (16). However, bone resorp-tion/remodeling of the maxilla after tooth loss often result in a residual ridge where primary stability can not be obtained (4). In these cases, bone-grafting procedures are often required in order to place implants in proper 3 dimensional positions. Autoge-nous block graft remains to be the gold standard for bone augmentation. The sites for harvesting the autogenous bone graft can be obtained either from intraoral such as ramus and chin or extraoral such as calvaria or iliac crest. While iliac crest belongs to endochondral origin bone, the other three mentioned are grafts of intramembranous origin. At this moment, there is no study that compares the implant stability outcome between these 2 distinct intramembraneous origin autogenous bone. Henceforth, the aim of study was to assess the ISQ on implants placed upon either ramus or calvaria bone blocks.

A quantitative measurement of implant stability, such as RFA, is essential prior to implant restoration. Stability is defined as “a measure of the difficulty of displacing an object or system from equilibrium” (17). Thus, implant stability could be considered as the absence of mobility, and this has been considered as the clinical meaning of the histologic term “osseointegration”. Many methods have been proposed to assess initial osseointegration (18). However, most of them are no longer available due to their invasiveness and inaccuracy (18). RFA used ISQ as a quantitative unit to assess implant stability has become a main tool these days for assessing implant stability (2). It is dependent of 3 main factors: (1) the stiffness of the implant fixture and its interface with the surrounding tissues, (2) the design of the transducer and (3) the total effective length above the bone level (19). It uses a small L-shape transducer that is tightened to the implant by a screw. This transducer comprises 2 piezoceramic elements, one vibrating by a sinusoidal sign (5 to 15 Hz) while the other serves as a receptor (20). It has been reported that RFA ISQ value ranged form 57 to 82 after 1 year of loading (3). Hence, values < 50 may be an assumption of potential risk of failure (20).

Results obtained from this study showed there was no significant difference on RFA ISQ value between implant placed on ramus and calvaria bone block. This suggests that intramembranous bone after healing, regardless of their locations, matures and converts in host bone. Additionally, both locations show to have the same ability to support implant placement. Intramembranous bone heals with thicker trabeculae and lower connectivity than endochondral origin bone (9). Hence, they show less resorption and higher revascularization when compared to endochondral origin bone (21). These reasons may explain the good behavior supporting implants providing them of high mechanical stability in order to achieve secondary implant stability. The present study shows that when they were incorporated into host bone in the attempt to support implant placement/stability, they behave similar (p= 0.154).

The correlation between implant diameter and RFA ISQ value was conducted and result demonstrated there was no correlation (p=138). This implies that as long as implant is integrated into bone no mater what diameter is, the reading remain to be similar. Since the RFA is used to detect implant stability, the finding does not surprise to us. In this study, we did not examine the influence of implant length upon RFA ISQ value since early studies have clearly demonstrated that as long as implant is integrated no matter what length is the value remained to be the same (15). Hence, no attempt was done in this experiment to look into this relationship. Even though, this is the first study that examined the correlation between implant diameters of implants placed in ridges grafted by autogenous block grafts and ISQ. Nonetheless, two studies looked at relationship between implant width and ISQ values, and both found that ISQ values were not influenced by the implant diameter (22,23) as observed in our study. Interestingly, implant width has been reported a major factor (more than length) to support prosthetic load (15). This is due to the role that plays the width of the coronal aspect of the implant in concentrating higher loads (15). It has been shown that increasing 0.5 mm in implant width provides 10%-15% more implant surface (15). Subsequently, it may be due to the greater bone-to-implant interface that achieves higher degree of osseointegration. Hence, it was assumed that wider implants provide higher ISQ values. However, our results and others contradict this assumption (22,23).

## Conclusion

RFA shows to be a reliable and noninvasive technique to foresee short-term implant stability in values over 70 for implants supported by bone block grafts. Furthermore, the origin of the intramembranous bone graft used for horizontal bone augmentation in the maxilla did not influence the outcome of ISQ values (p=0.154). In addition, no correlation was established between implant diameter and ISQ value (p= 0.138).
